# Supplementary material for: Best Practice PD-L1 Staining and Interpretation in Gastric Cancer Using PD-L1 IHC PharmDx 22C3 and PD-L1 IHC PharmDx 28-8 Assays, with Reference to Common Issues and Solutions
Source: Biomedicines. 2025 Nov 19;13(11):2824. doi: 10.3390/biomedicines13112824 (PMC12649831; doi:10.3390/biomedicines13112824)
Supplement: Supplementary file 1 [file biomedicines-13-02824-s001.zip › biomedicines-3927145-supplementary.pdf]

Supplementary Table S1. PD-L1 Laboratory Checklist for Gastric Cancer

Step	Checklist Item	Key Points / Recommendations
1. Specimen adequacy (Pre-analytical)	Minimum tumor content	At least 100 viable tumor cells should be present for valid PD-L1 evaluation.
	Fixation and embedding	Formalin-fixed, paraffin-embedded (FFPE) tissue with 6–48 h fixation in 10% neutral buffered formalin.
	Block age	Use recent FFPE blocks (<3 years old when possible)
	Section storage	Cut sections 4–5 µm; store at 2–8 °C and stain within manufacturer's recommended period (e.g., ≤5 months for 22C3).
2. Analytical phase	Assay selection	Use FDA-approved companion diagnostic assays (e.g., PD-L1 IHC 22C3, 28-8)
	Platform verification	Ensure platform compatibility (Autostainer Link 48, Benchmark, etc.) and validated staining protocol.
	Controls	Include on-slide positive and negative control tissues for each batch.
3. Interpretation and reporting (Post-analytical)	Scoring system	Use Combined Positive Score (CPS = [PD-L1+ tumor + PD-L1+ immune cells]/total tumor cells ×100).
	Exclude from CPS count	• Cytoplasmic-only tumor staining<br>• PD-L1+ normal glands/metaplasia<br>• Smooth muscle, fibroblasts, endothelial cells<br>• Neutrophils/plasma cells/ eosinophils<br>• Necrotic/mucinous debris
	Confirm with H&E	Verify PD-L1+ cells correspond to viable tumor or mononuclear immune cells on H&E.

	Reporting details	Specify assay clone, platform, cutoff (e.g., CPS ≥1, ≥5), sample type (biopsy/resection), and adequacy statement.
4. Quality assurance	Internal QC	Monitor inter-run and inter-observer concordance; document deviations.
	External QA	Participate in external proficiency testing or EQA programs when available.
	Documentation	Record reagent lot numbers, control performance, and review log for all cases.
